# Synthesis and Characterization of a Nearly Single Bulk Ti_2_AlN MAX Phase Obtained from Ti/AlN Powder Mixture through Spark Plasma Sintering

**DOI:** 10.3390/ma14092217

**Published:** 2021-04-26

**Authors:** Christopher Salvo, Ernesto Chicardi, Rosalía Poyato, Cristina García-Garrido, José Antonio Jiménez, Cristina López-Pernía, Pablo Tobosque, Ramalinga Viswanathan Mangalaraja

**Affiliations:** 1 Departamento de Ingeniería Mecánica, Facultad de Ingeniería, Universidad del Bío-Bío, Concepción 4081112, Chile; 2 Departamento de Ingeniería y Ciencia de Materiales y del Transporte, Universidad de Sevilla, 41092 Sevilla, Spain; echicardi@us.es; 3 Instituto de Ciencia de Materiales de Sevilla (ICMSE-CSIC), 41092 Sevilla, Spain; rpoyato@us.es; 4 Instituto Andaluz del Patrimonio Histórico (IAPH), Camino de los Descubrimientos s/n., 41092 Sevilla, Spain; cristina.g.garrido@juntadeandalucia.es; 5 Centro Nacional de Investigaciones Metalúrgicas, Consejo Superior de Investigaciones Científicas (CENIM-CSIC), 28040 Madrid, Spain; jimenez@cenim.csic.es; 6 Departamento de Física de la Materia Condensada, ICMS, CSIC-Universidad de Sevilla, 410902 Sevilla, Spain; cristinalopez@us.es; 7 Laboratorio de Películas Delgadas y Procesos Electroquímicos, Departamento de Ingeniería de Materiales, Universidad de Concepción, Concepción 4070386, Chile; pablotobosque@udec.cl; 8 Laboratorio de Nanotecnología y Cerámicos Avanzados, Departamento de Ingeniería de Materiales, Universidad de Concepción, Concepción 4070386, Chile

**Keywords:** MAX phase, Ti_2_AlN, spark plasma sintering

## Abstract

MAX phases are an advanced class of ceramics based on ternary carbides or nitrides that combine some of the ceramic and metallic properties, which make them potential candidate materials for many engineering applications under severe conditions. The present work reports the successful synthesis of nearly single bulk Ti_2_AlN MAX phase (>98% purity) through solid-state reaction and from a Ti and AlN powder mixture in a molar ratio of 2:1 as starting materials. The mixture of Ti and AlN powders was subjected to reactive spark plasma sintering (SPS) under 30 MPa at 1200 °C and 1300 °C for 10 min in a vacuum atmosphere. It was found that the massive formation of Al_2_O_3_ particles at the grain boundaries during sintering inhibits the development of the Ti_2_AlN MAX phase in the outer zone of the samples. The effect of sintering temperature on the microstructure and mechanical properties of the Ti_2_AlN MAX phase was investigated and discussed.

## 1. Introduction

MAX phases are a relatively new class of advanced ceramics that adopted this name due to the general formula that describes them, M_n+1_AX_n_, where *n* = 1, 2, or 3, M is an early transition metal, A is an IIIA/IVA-group element and X is C and/or N. These ternary carbides or nitrides combine some of the properties of ceramics and metals, exhibiting high electrical and thermal conductivities, good thermal shock resistance, superior oxidation/corrosion resistance and ease of machining [1,2]. These combined properties, which emanate from their atomic bonding and the nature of the crystalline structure, make them potential candidate materials to be used in advanced technological applications under severe conditions, such as high temperature, aggressive corrosion environment, and high irradiation in aerospace or nuclear systems [3,4,5].

MAX phases have a layered hexagonal crystal structure with P63/mmc space group symmetry, which consists of alternative near close-packed layers of M_6_X octahedral (being connected between each similar layer by shared edges) and the layers of A atoms located at the center of the trigonal prisms [3]. The different MAX stoichiometries are classified according to the number of M layers separated by the A layers: M_2_AX (211), M_3_AX_2_ (312), and M_4_AX_3_ (413) in which there are two, three, and four, respectively [6]. Although more than 155 MAX phases have been reported [7], the MAX phases based on the Ti-Al-N and Ti-Al-C ternary systems are particularly attractive for industrialization since these are some of the most lightweight and oxidation resistant materials [8]. The synthesis of a single-phase bulk material of Ti_2_AlN is difficult due to the reduced stability zone in the Ti-Al-N ternary system [9,10] which is less investigated, despite exhibiting better properties than the other MAX phases due to its stronger chemical bonding [11].

There are several powder metallurgy methods to synthesize the bulk Ti_2_AlN MAX phase, starting with the combination of Ti/Al (using N_2_ atmosphere), Ti/AlN or Ti/Al/TiN as raw materials, including hot isostatic pressing (HIP) [12,13], self-propagating high-temperature synthesis (SHS) [14,15,16,17], mechanical alloying (MA) and hot pressing (HP) [18], SHS and HP [19], HP [20,21,22,23], reaction sintering [24,25] and spark plasma sintering (SPS) [26,27,28,29,30]. Among them, the SPS demonstrates economic and technological advantages in the processing and formation of single phases, such as shorter soaking time, lower sintering temperatures, higher density and densification rate, improved mechanical properties, and refined grain sizes [31]. The SPS is a field-assisted sintering technique (FAST) where a uniaxial compaction pressure is applied to the sample along with a pulse direct current to obtain the fully dense and nearly single phase of MAX phases from milled or unmilled powders [31,32]. The microstructure and phases in the final samples are significantly influenced by the SPS processing parameters [33]. Akhlaghi et al. [34] claimed that a very high heating rate of the SPS is an essential requirement for the single MAX phase synthesis.

The previous reports of the Ti_2_AlN MAX phase obtained by the SPS method also turned out to be accompanied by secondary undesired phases, reaching up to 10 wt. % of these, which depended mainly on the consolidation parameters and starting powders [28,29,35]. Therefore, there is still a lack of knowledge about the optimum parameters for obtaining a single bulk Ti_2_AlN MAX phase. The present work is focused on the synthesis of the bulk Ti_2_AlN MAX phase and the prevention of secondary undesired phase formation by the optimization of the SPS consolidation parameters.

## 2. Materials and Methods

The powders used as the raw materials in this work, such as AlN (325 mesh, CAS number 24304-00-5, O: 7500 ppm, C: 300 ppm, and N: 33.47%) and Ti (99.5% purity, -325 mesh, CAS number 7440-32-6, O: 0.22%, C: 0.01% and N: 0.01%), were purchased from Alfa Aesar, Haverill, MA, USA. A mixture of the Ti and AlN starting powders was prepared in a molar ratio of 2:1 by mixing in a Turbula^®^ T2F mixer for 4 h. The observation of particle morphology of the raw powders and the Ti:AlN powders mixture was carried out using a JEOL scanning electron microscope (SEM) model JSM-6380LV 9 (JEOL, Peabody, MA, USA). The reactive sintering of the powder mixtures was carried out by using the spark plasma sintering (SPS) technique in vacuum atmosphere (10 Pa). The powder mixture was introduced in a graphite die with an inner diameter of 15 mm and placed into the spark plasma sintering device (Model 515S, SPS Sinter Inc. Kanagawa, Japan, CITIUS). The reactive sintering was carried out with a heating rate of 100 °C/min up to the sintering temperature of 1200 °C and 1300 °C for 10 min under 30 MPa of uniaxial pressure. The temperature was measured with an optical pyrometer (SPS Sinter Inc. Kanagawa, Japan) by focusing on the side of the graphite die. The resulting samples were denoted as MAX-1200 and MAX-1300 for the powder mixtures consolidated at 1200 °C and 1300 °C, respectively. The experimental absolute density of the pellet samples was determined by the Archimedes method according to the ASTM B962-17 standard [36] and the percentage of densification was also calculated by considering the theoretical density of Ti_2_AlN, which is 4.30 g/cm^3^. The microhardness values of the sintered samples were obtained using a Vickers diamond pyramidal indenter, Zwick 3212 (Zwick, Ulm, Germany), with an applied load of 10 N for 10 s. In this test, ten indentations were made on each sample at room temperature. The Vickers hardness was determined by the ratio of the applied load via a geometrically defined indenter to the contact (projected) area of the resultant impression, using the following Equation (1):

(1) $$\mathrm{HV}=1854.4\;\frac{P}{d^{2}}$$

where *P* is the applied load (kg) and *d* is the indentation diagonal length (mm).

The X-ray diffraction (XRD) measurements were carried out to characterize the crystalline phases present in the samples by using a Bruker AXS D4 Endeavor diffractometer with Cu-K_α_ radiation (Bruker AXS, Karlsruhe, Germany). The XRD data were recorded in the conventional Bragg–Brentano geometry for 2θ scans ranging from 30° to 90° with a step width of 0.02° and a counting time of 2 s/step. A current of 20 mA and a voltage of 40 kV were employed as tube settings. The crystalline phases present in the XRD patterns were determined using the DIFFRACplus EVA software by Bruker AXS and the JCPDS database. The microstructural information and phase quantification were performed by fitting the whole measured diffraction pattern with version 4.2 of the TOPAS (Bruker AXS) Rietveld analysis software. The crystallographic information was obtained from Pearson’s crystal structure database [37]. The microstructure was analyzed on both polished and fractured surfaces of the sintered samples using a HITACHI field emission scanning electron microscope (FESEM) (Hitachi Co., Ltd., Tokyo, Japan), model S-4800, equipped with a Bruker-XFlash 4010 energy dispersive X-ray spectrometer (EDS). Moreover, the mean grain size was determined from the fractured samples by image analysis using the Image-Pro Plus software.

## 3. Results and Discussions

**Figure 1:**
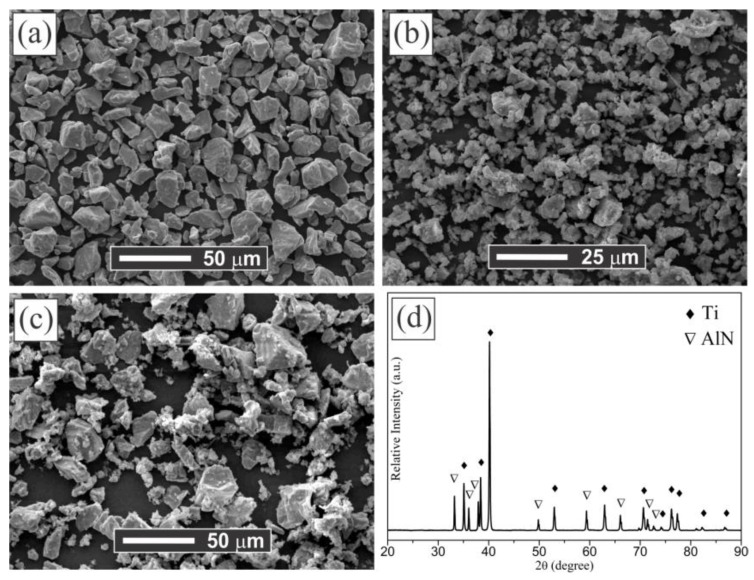
(**a**,**b**) SEM images for the Ti and AlN as-received powders, respectively; (**c**,**d**) SEM image and XRD of the Ti:AlN powder mixture, respectively.

The morphology and size of the raw Ti and AlN powders are shown in the SEM images of Figure 1a,b, respectively. It is observed that the angular morphology of Ti and AlN powders have an average particle size around 20 and 10 µm, respectively. Moreover, the Ti and AlN powder mixture is shown in the SEM micrograph of Figure 1c and its corresponding XRD pattern is presented in Figure 1d. The phases present in this diffraction pattern are identified by the search–match technique using the JCPDS database, which exhibited the peaks that are corresponded to Ti (JCPDS No.: 44-1294) and AlN (JCPDS No.: 25-1133) and showed the absence of any additional phase.

**Figure 2:**
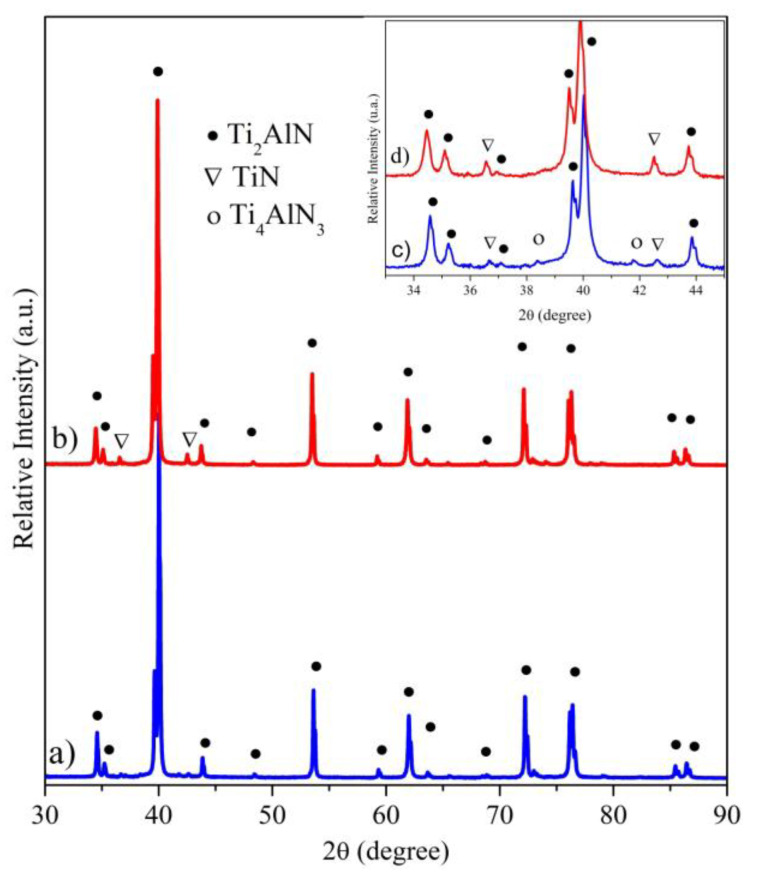
XRD patterns of the bulk Ti_2_AlN MAX phase samples: (**a**) MAX-1200 (blue pattern) and (**b**) MAX-1300 (red pattern). A zoom of the XRD patterns between 2θ = 33° and 45° for (**c**) MAX-1200 and (**d**) MAX-1300.

The X-ray diffraction (XRD) patterns of the MAX-1200 and MAX-1300 samples obtained by reactive spark plasma sintering (SPS) of the Ti and AlN powder mixture are shown in Figure 2. Moreover, a zoom of the XRD patterns between 33° and 45° is presented on the upper right part of Figure 2. It is observed that both samples mainly consisted of the Ti_2_AlN MAX phase (JCPDS No.:18-0070), those peaks have the highest relative intensity. However, the XRD of the samples evidenced the presence of the slight diffraction peaks corresponding to TiN (JCPDS No.:38-1420). The relative intensity of the diffraction peaks associated with the TiN is increased with the sintering temperature, which is associated with a higher relative quantity. Moreover, the sample MAX-1200 exhibited diffraction peaks that are associated with the Ti_4_AlN_3_ MAX phase (JCPDS No.:65-9771). Nevertheless, the low relative intensity of these peaks suggested a very small amount of undesired phases. The Rietveld analysis determined the amounts lower than ~2 wt. % of ancillary phases for the MAX-1200 and MAX-1300 samples, reaching a nearly single-phase bulk Ti_2_AlN (>98% purity). The reduced amounts of the ancillary phases are associated with an intermediate phase for the case of the TiN that is related to the formation mechanism of the Ti_2_AlN MAX phase [23]. On the other hand, the Ti_4_AlN_3_ corresponds to a high-order MAX phase and its production and the mechanism of the formation have been scarcely reported [35,38,39]. It is observed that by increasing the sintering temperature, the XRD peaks of the Ti_4_AlN_3_ disappeared and, simultaneously, the relative intensity of the TiN peaks increased. This is associated with the decomposition of the Ti_4_AlN_3,_ whose process is based on the formation of TiN_0.75_ via sublimation or de-intercalation of Al. However, previous studies reported that it occurred slowly at 1450 °C under vacuum [40].

It is expected that a solid-state reaction between Ti and AlN generates the Ti_2_AlN MAX phase under several associated steps. In this case, the mechanism of formation and the reaction sequences between 800 °C and 1450 °C that occur during synthesis by reactive SPS of Ti and AlN powder mixtures have been previously discussed in an earlier report [28], where nearly a single bulk Ti_2_AlN MAX phase was obtained at 1400 °C under 50 MPa of uniaxial pressure for 5 min of dwell time.

Recently, Gilev and Kachenyuk [29] have reported the phase formation in the synthesis of Ti_2_AlN by SPS using Ti and AlN powders (mechanically activated) as the starting materials over the temperature range of 900–1400 °C, where the maximum purity (90%) of the Ti_2_AlN MAX phase was achieved at 1300 °C under 10–30 MPa of uniaxial pressure for 5 min of dwell time. In comparison, it is remarkable that we achieved nearly a single-phase bulk Ti_2_AlN MAX (>98% purity) starting from the Ti/AlN powder mixture at the lower sintering temperature (1200 °C) and processed it through reactive SPS. This is mainly attributed to the experimental procedure’s differences, such as: (a) the absence of mechanical activation process before sintering; (b) double dwell time; and (c) the lower uniaxial pressure used. It is known that the ball-milling process carried out before the sintering stage promoted a grain fragmentation and increased the microstrain [26], which has been reported in the case of Ti_2_AlN to induce the formation of undesired secondary phases [25,41]. The Ti-Al-N ternary phase diagram is a difficult system to study due to the coexistence of a wide variety of phases in the different temperatures at which the Ti_2_AlN is stable [9,10], which is observed between 700 °C and 1600 °C [42]. Moreover, the phases like TiN and TiAl are the byproducts that can be obtained during the synthesis of MAX phases and therefore frequently coexist with them [26]. On the other hand, Yan et al. and Cui et al. [26,27] have obtained nearly a single-phase bulk Ti_2_AlN through SPS at 1200 °C and 1300 °C, respectively, but from the starting powders of Ti:Al:TiN in a molar ratio of 1:1:1 and 1:1.1:1, respectively. Similarly, Li et al. [30] reported the synthesis of a nearly pure Ti_2_AlN by using different Al molar ratios, focusing on the significant role in the improvement of purity that small amounts of Al exhibited; however, any further excess of aluminum resulted in the appearance of Ti_x_Al_y_ ancillary phases.

**Figure 3:**
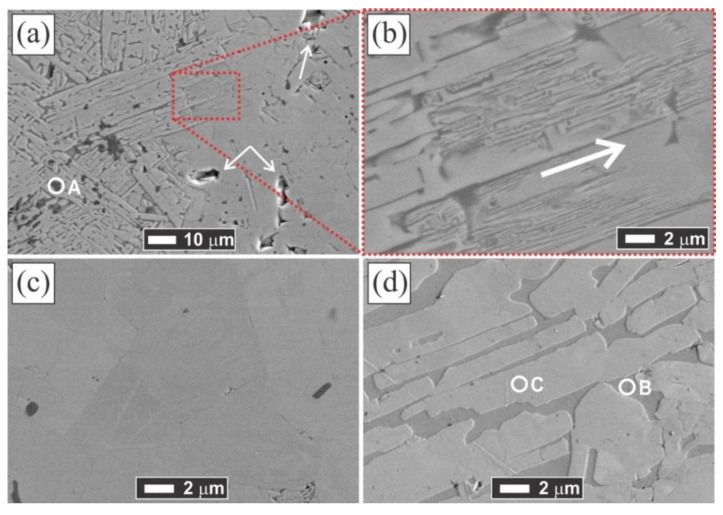
SEM images of: (**a**) the outer border of the polished and non-etched MAX-1200 sample; (**b**) zoom of the “transition zone”; (**c**) the middle of the sample (showing a nearly single bulk Ti_2_AlN MAX phase); and (**d**) underdeveloped Ti_2_AlN. The EDS point analysis carried out revealed that the zones A, B, and C correspond to Al_2_O_3_, Ti_3_Al, and Ti_4_AlN_3_, respectively.

**Figure 4:**
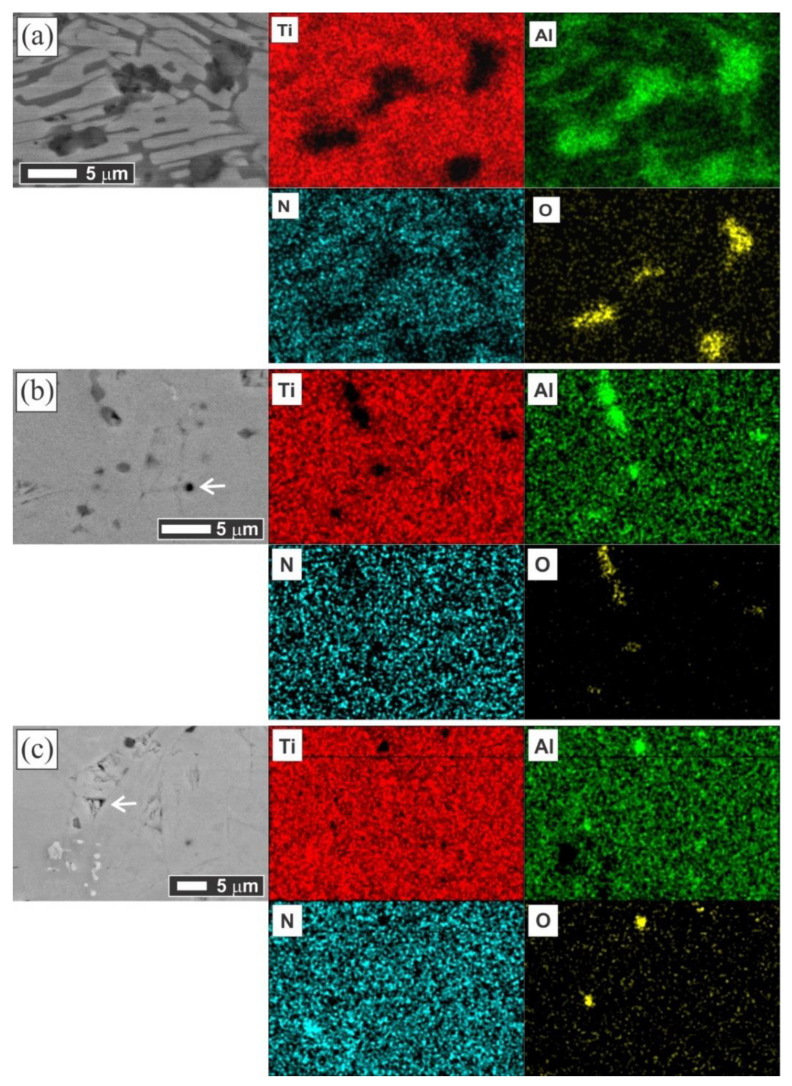
SEM images of the polished and non-etched samples with the elemental maps of the same area for the Ti (red), Al (light green), N (light blue) and O (yellow) for the (**a**) outer border zone of MAX-1200, (**b**) MAX-1200 middle zone and (**c**) MAX-1300 middle zone.

To characterize the microstructure of the sintered samples, the SEM images were acquired, and their compositions were observed by EDS. The fact of the existence of other phases in the lower amounts than the XRD sensitivity limit detection and the sensitive composition range of Ti_2_AlN is exposed in Figure 3a. This SEM image has been taken at a point on the outer border of the MAX-1200 sample, and the existence of two different microstructures is indicated. For a better understanding, the higher magnification SEM images of Figure 3a are shown for the microstructure of a nearly single-phase Ti_2_AlN (Figure 3c) and the underdeveloped Ti_2_AlN (Figure 3d). The EDS point analysis revealed the composition of the Ti_2_AlN phase and also an elemental map zone of this microstructure is shown in Figure 4a) which are: (i) the dark phase (A point, Figure 3a) shows an absence of N, and its Ti:Al:O elemental ratios are: 4.9 ± 0.3 at.% Ti, 33.8 ± 0.7 at.% Al and 61.2 ± 1.1 at.% O; (ii) the grey phase (B point on Figure 3d) shows an absence of N and O, and its Ti:Al elemental ratios are 73.4 ± 1.5 at.% Ti, and 26.6 ± 0.9 at.% Al and; (iii) the white phase (C point, Figure 3d) shows that its Ti:Al:N elemental ratios are 50.9 ± 2.2 at.% Ti, 9.5 ± 0.4 at.% Al, and 39.6 ± 2.7 at.% N, very near to the “4:1:3” MAX stoichiometry. This suggested that the dark phase corresponded to the Al_2_O_3_ particles which are embedded between elongated/platelike Ti_4_AlN_3_ phase grains (white phase) and intermetallic Ti_3_Al phase (grey phase). It is supposed that this unexpected mixed microstructure (Ti_4_AlN_3_+Ti_3_Al+Al_2_O_3_) is generated during the formation of the Ti_2_AlN MAX phase. The formation of Ti_2_AlN through Ti and AlN powders has been previously described. Concretely, when the Al and N atoms of AlN have enough energy, they diffuse into the Ti. Then, TiN and Ti-Al compounds are formed [22], together with a variety of others Ti-Al phases such as TiAl, Ti_3_Al, Al_3_Ti and, Al_2_Ti. Finally, due to the solubility of the N atoms, TiN and TiAl react and precipitate as Ti_2_AlN [5,20]. The presence of the Ti_4_AlN_3_ MAX phase has not exhibited concordance with the previous reports and its formation is attributed to the conditions that occurred only at the border of the sample, in which a high amount of Al was consumed for the massive formation of Al_2_O_3_ particles. Previous studies reported a thermally activated diffusion mechanism to explain the formation of Ti_4_AlN_3_, which is based on a transformation from a MAX phase of a low *n* order to a higher-order [39]. The grains observed of the Ti_4_AlN_3_ are similar to the ones obtained by Barsoum et al. [38] who reported for the first time the synthesis of Ti_4_AlN_3_, which was obtained by HIP using the starting powders of TiH_2_, AlN, and TiN at 1275 °C for 24 h under 70 MPa. Further microstructural studies are required for the scientific community to deeply understand this behavior.

On the other hand, the EDS analysis of the right zone (a Ti_2_AlN single-phase) of Figure 3a shows that its Ti:Al:N elemental ratios are 51.4 ± 1.4 at.% Ti, 21.6 ± 1.5 at.% Al, and 27.0 ± 2.5 at.% N, which corresponds to the Ti_2_AlN MAX phase. Additionally, a visible grain chipping is observed in this zone (indicated with white arrows), which may occur during the polishing step.

Moreover, the layered-structured Ti_2_AlN formation progress by comparing the microstructures shown in Figure 3b,d, observing that the laminated microstructure is finer as the Ti:Al:N atomic ratio approaches 2:1:1 (indicated with the white arrow). The Al_2_O_3_ particles presented a size of around 5 µm and they are heterogeneously distributed, mainly located between the Ti_4_AlN_3_ phase grains or inside them (Figure 3a and Figure 4a). Then, it is concluded that the Ti_2_AlN formation is inhibited due to the formation of the Al_2_O_3_ particles in the border of the sample. The calculated value for the partial pressure of oxygen at the vacuum used (10 Pa) by Dalton’s law corresponds to 2.1 Pa, which is much higher than the partial pressure of oxygen in equilibrium at 1200 °C (10^−23^ Pa), which may have caused the oxidation of the metal [43], preferably at the border of the sample. Then, it would be reasonable to think about using a higher vacuum than the used one or some inert gas for the synthesis of a higher purity of this material. The vapor pressure values for Ti and Al at the optimum formation temperature of Ti_2_AlN MAX phase (1200–1300 °C) corresponded to 5 × 10^−5^ and 0.8 Pa, respectively, indicating that the use of high vacuum should volatilize and sublime the Al and some Ti, then preferring the use of some inert gas (argon or helium) atmosphere for the reactive sintering of Ti_2_AlN.

The elemental maps of the microstructures of both MAX-1200 and MAX-1300 samples are shown in Figure 4b,c, respectively, which have been taken in the middle part of the samples. There are some small Al_2_O_3_ inclusions (dark phase) of around 2 µm are observed in both bulk Ti_2_AlN MAX phase samples. This kind of inclusion has been reported several times during the formation of the Ti_2_AlN MAX phase, which can be attributed to the presence of oxygen in the raw powders [12,44,45]. The EDS analysis carried out in the microstructure matrix (grey phase) confirmed the atomic ratio of Ti:Al:N is 2:1:1, indicating that the grey phase (main phase) of Figure 4b,c is Ti_2_AlN. Besides, the isolated particles of TiN (white phase) are observed in the bulk Ti_2_AlN which was sintered at 1300 °C (Figure 4c).

**Figure 5:**
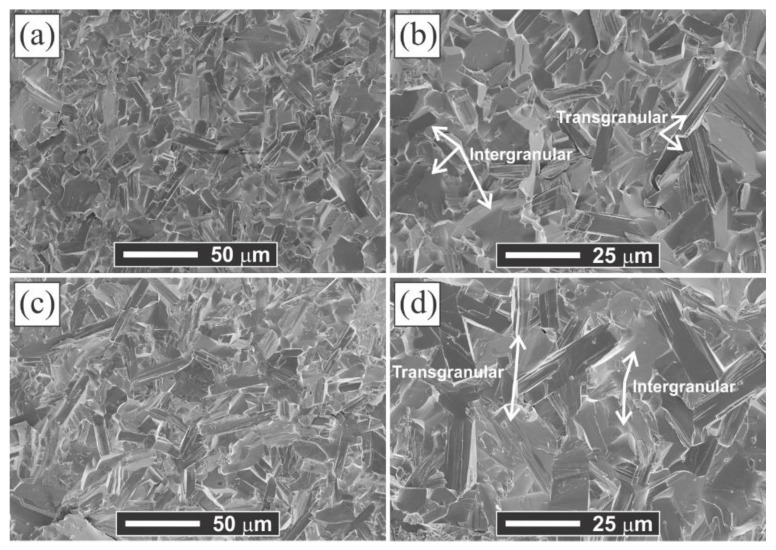
SEM images of the fracture surfaces for (**a**,**b**) MAX-1200 and (**c**,**d**) MAX-1300.

The fracture surfaces of the MAX-1200 and MAX-1300 samples are shown in Figure 5a–d, respectively. The fracture mechanism is a combination of transgranular and intergranular cleavage, the usual fracture mechanism for ceramics. Concretely, the intergranular and transgranular fractures are observed (some are pointed out with white arrows in Figure 4b,d), and the transgranular fracture represented the layered-grain structural character which is typical for the MAX phases. The fracture surfaces revealed a mixed grain morphology with equiaxed and platelike grains. The latter showed a rapid grain growth (around double) by increasing the sintering temperature from 1200 (MAX-1200) to 1300 °C (MAX-1300), passing the platelike grains from ~18 and 5 µm (mean length and width size) to ~30 and 10 µm, respectively. To study this aspect, we calculated the average grain aspect ratio of the samples produced at 1200 and 1300 °C, which corresponded to 3.3 and 3.1, respectively. These ratios are similar, which may indicate a non-preferential direction growing of the grains of Ti_2_AlN. Cui et al. [26] reported an average grain aspect ratio of 3.7 for Ti_2_AlN produced from the starting mixtures of Ti/Al/TiN by SPS.

The bulk relative density measured by the Archimedes principle by the immersion in water of both MAX-1200 and MAX-1300 samples is very similar and reached ~97% of the theoretical density. According to the literature, obtaining complete densification in the Ti_2_AlN MAX phase by SPS is difficult. The density values are not clearly reported and the few published reports only mentioned that the samples obtained are nearly fully dense. Cui et al. reported densification of 99.0% using a mixture of Ti/Al/TiN consolidated by the reactive SPS [26] and Ming et al. reported densification of 97.9% using a mixture of Ti/Al/TiN consolidated by the reactive hot pressing [21]. It seems that the only way to obtain the fully dense Ti_2_AlN MAX phase is using a very long consolidation time, as Barsoum et al. reported (1400 °C and 40 MPa during 48 h using the reactive HIP) [12], but assuming the disadvantage of having between 10 and 15 wt.% of ancillary phases and the higher cost associated. The hardness values obtained for the MAX-1200 and MAX-1300 samples corresponded to 4.4 ± 0.1 and 4.0 ± 0.1 GPa, respectively. By comparing with their respective grain sizes, it is noticed that the hardness depended on the mean grain size in the Ti_2_AlN MAX phase, which followed the Hall–Petch relation. Moreover, these values are in accordance with the range of the values reported previously obtained under different routes, such as 4.0, 4.3, and 3.5 GPa, respectively, [12,20,23], which remarked that the processing route or initial precursor powders used to obtain the Ti_2_AlN may not have influenced in the final mechanical properties of the material.

## 4. Conclusions

The nearly single bulk Ti_2_AlN MAX phase (>98% purity) was successfully prepared from the Ti:AlN powder mixture in a molar ratio of 2:1, respectively, by the reactive spark plasma sintering at 1200 and 1300 °C. The importance of using non-mechanically activated powders to obtain a single Ti_2_AlN MAX phase was noted. The final microstructure was composed of equiaxed and elongated platelike grains and the formation mechanism of the interlaminated microstructure, typical of the MAX phases, was revealed by the inhibition of Ti_2_AlN development due to the massive formation of Al_2_O_3_ particles in a small zone in the border of the MAX-1200 sample. The densification of both MAX-1200 and MAX-1300 samples was very similar and reached ~97% of the theoretical density, and their hardness values were 4.4 and 4.0 GPa, respectively.

## Data Availability

The data presented in this study are available on request from the corresponding author.
